# Elite US college admissions: could the quest for admission increase overuse injury risk?

**DOI:** 10.5249/jivr.v8i2.791

**Published:** 2016-07

**Authors:** David C. Schwebel, Jingzhen Yang

**Affiliations:** ^*a*^Department of Psychology, University of Alabama at Birmingham, Birmingham, USA.; ^*b*^Center for Injury Research and Policy, Nationwide Children’s Hospital, Columbus, OH, USA.

## Abstract

This commentary addresses the intriguing correspondence of two trends. First, we describe the increasing selectivity for undergraduate admission to elite colleges and universities in the United States and an apparent preference for “angular” applicants who have demonstrated tremendous accomplishment in a single non-academic pursuit such as music, athletics, or the arts. Second, we describe an apparent increase in overuse injuries among American children and adolescents, a trend that many experts attribute to specialization within a single athletic, musical or artistic pursuit among youth who in previous generations were more “generalist” in their extracurricular activities. It is premature to demonstrate causality and suggest increasing college selectivity has led to increasing rates of overuse injuries, but we speculate there may be a causal relation present and encourage scholarly research on the topic.

## Introduction

Over the last two decades, the United States (US) college admissions process has changed dramatically. Admissions rates at elite American colleges have plummeted, reaching lows of 5-8% at selective colleges and universities in recent years.^1,2^ In response to these trends, promising high school students have searched for ways to distinguish themselves, attempting to prove they would be more successful than the thousands of other applicants they compete with for entry into selective colleges and universities.

One consequence of these trends is the apparent desire of elite colleges in the United States to admit so-called “angular” applicants, those who have demonstrated tremendous accomplishment in a single non-academic pursuit such as music, athletics, or the arts.^3-4^ It is not entirely clear why elite college admissions trends have moved from preferring the accomplished well-rounded applicant to the accomplished angular applicant. Some argue it allows universities to boast about their successful students, who achieve national prominence in particular domains.^3^ Others claim that it is simply a way to distinguish students for admittance among a tremendously gifted set of applicants.^4^ Whatever the reason, experts offer universal agreement that angular applicants have a bit of an edge for admissions to most elite US colleges and universities.

Over the past two decades, another trend has emerged in what appears to be a very different domain: overuse injuries among American children and adolescents.^5-6^ Although data are difficult to obtain and definitions of “overuse injury” differ widely, there is reasonable scholarly consensus that the repetitive motion involved in playing a single youth sport for prolonged hours on multiple days per week, often year-round and with minimal rest, contributes to high levels of muscle strain and consequential risk of overuse injuries.^5-7^ Risk is most prominently reported from athletic pursuits but is found also in other domains, including dance and playing stringed instruments.^8^ Early sport specialization at a very young age rather than “generalist” athletic participation in diverse year-round sports, may be causing an increase in overuse injury rates among American youth.^6-9^

Is it possible that overuse injury incidents are increasing, at least in small part, because youth are specializing into single pursuits with the goal of becoming the angular applicant admitted to Harvard, Stanford, or Yale? Is the previous 3-sport varsity letterman now playing baseball year-round? Is the previous young artist who excelled in ballet, violin, and choir now playing violin exclusively? If so, are American youth injuring themselves through overuse, pushed too far just to gain entry into the “right” college?

Demonstrating causality is premature and overuse injury data remain imprecise, but the overlapping trends cause pause.

## Acknowledgements

Thanks to Dawn Comstock for helpful feedback on the manuscript.
